# Transcriptomic Analysis of Genes Associated with Oxidative Stress in Chronic Rhinosinusitis Patients with Nasal Polyps: Identifying Novel Genes Involved in Nasal Polyposis

**DOI:** 10.3390/antiox11101899

**Published:** 2022-09-25

**Authors:** Yih-Jeng Tsai, Yu-Ting Hsu, Ming-Chieh Ma, Chun-Kuang Wu, Sheng-Dean Luo, Wen-Bin Wu

**Affiliations:** 1 Department of Otolaryngology Head and Neck Surgery, Shin Kong Wu Ho-Su Memorial Hospital, Taipei 111045, Taiwan; 2 School of Medicine, Fu Jen Catholic University, New Taipei City 242062, Taiwan; 3 Department of Otolaryngology Head and Neck Surgery, Cheng-Ching Hospital, Chung Kang Branch, Taichung 407211, Taiwan; 4 Department of Finance and International Business, College of Management, Fu Jen Catholic University, New Taipei City 242062, Taiwan; 5 Department of Otolaryngology, Kaohsiung Chang Gung Memorial Hospital and Chang Gung University, College of Medicine, Kaohsiung 833253, Taiwan; 6 Graduate Institute of Clinical Medical Sciences, College of Medicine, Chang Gung University, Taoyuan 33302, Taiwan; 7 Graduate Institute of Biomedical and Pharmaceutical Science, Fu Jen Catholic University, New Taipei City 242062, Taiwan

**Keywords:** CRS, CRSwNP, lactoperoxidase, oxidative stress, ROS, transcriptional profiling

## Abstract

Chronic rhinosinusitis with nasal polyps (CRSwNP) is a complicated inflammatory disease, and the underlying mechanism remains unclear. While some reactive oxygen/nitrogen species-related gene products are reported to participate in CRSwNP, a systemic and full analysis of oxidative-stress-associated genes in CRSwNP has not been extensively studied. Therefore, this study sought to catalog the gene-expression patterns related to oxidative stress and antioxidant defense in control and CRSwNP patients. In total, 25 control and 25 CRSwNP patients were recruited. The distribution and expression of 4-hydroxynonenal and 3-nitrotyrosine as markers of oxidative stress—which is represented by lipid peroxidation and the protein nitration of tyrosine residues in CRSwNP nasal polyps (NPs)—were more apparently increased than those found in the control nasal mucosae, as determined by immunohistochemistry (IHC). The expression of 84 oxidative-stress-related genes in nasal mucosae and NP tissues was analyzed via real-time PCR, which showed that 19 genes and 4 genes were significantly up- and downregulated, respectively; among them, inducible nitric oxide synthase (iNOS) and heme oxygenase 1 (HO-1) were notably upregulated, whereas lactoperoxidase (LPO), myeloperoxidase (MPO), and superoxide dismutase 3 (SOD3) were highly downregulated. Changes in the mRNA and protein levels of these redox proteins were confirmed with a customized, real-time PCR array and RT-PCR analysis, as well as Western blotting and IHC assays. A receiver operating characteristic curve analysis further suggested that LPO, MPO, SOD3, HO-1, and iNOS are possible endotype predictors of CRSwNP development. Collectively, we present an oxidative-stress-related gene profile of CRSwNP NP tissues, providing evidence that the systemic changes in oxidative stress and the antioxidative defense system, including novel iNOS, heme peroxidases, and other genes, are closely linked to CRSwNP pathology, development, and progression.

## 1. Introduction

Chronic rhinosinusitis (CRS) affects people of all ages and is one of the most prevalent chronic illnesses in the United States and countries worldwide. The disease affects 12.5% of the population worldwide and more than about 10% of the Asian population [1,2,3]. CRS can be subdivided into two major categories based on whether nasal polyps (NPs) are present or absent, namely, CRS with nasal polyps (CRSwNP) and CRS without nasal polyps (CRSsNP), which is characterized by the presence of various degrees of tissue remodeling [4,5]. The burden of CRS is high, especially in the CRSwNP phenotype [6,7]. Moreover, in most cases, patients with CRSwNP are considered to have more clinically severe symptoms and signs than those without NPs [8]. Surveys based on patient self-reports have demonstrated that CRSwNP adversely influences various aspects of quality of life and have a more unfavorable effect on social functioning than many other chronic diseases [9]. Therefore, a better understanding of CRSwNP pathogenesis and pathophysiology is needed to advance the current diagnostic and treatment strategies available to affected patients.

Oxidative stress refers to the excessive production of reactive oxygen/nitrogen species (ROS/RNS) in cells and tissues, in which an antioxidant system cannot neutralize them [3]. The accumulation of ROS/RNS-induced damage is responsible for the development of diseases, most of which are chronic inflammatory diseases [10]. This includes damage to cellular molecules such as DNA, proteins, and lipids [3] and the possible recruitment of macrophages and leucocytes to cause a form of inflammation called a “respiratory burst” [11]. Oxidative stress is a defense mechanism in nasal mucosae that directly damages bacterial infections. It has been shown that lipids, such as cholesteryl linoleate and arachidonate, in nasal epithelial membranes can contribute to the antimicrobial properties of nasal secretions, and their levels can increase in the nasal secretions in CRS patients [12]. However, the imbalance between ROS/RNS production and antioxidant defense systems can cause damage via oxidative stress, including oxidized proteins, glycated products, and lipid peroxidation, resulting in inflammation, neuron degeneration in brain disorders, somatic mutations, and even neoplastic transformations/malignancies [11,13].

It was reported that the levels of oxidative stress in nasal secretions, in the forms of total antioxidant status, malondialdehyde, and nitric oxide (NO), correlate with the severity of the nasal obstruction and congestion in CRS nasal polyposis in Turkish people [14]. In addition, in dual oxidases (DUOX1 and 2), which are responsible for H_2_O_2_ generation in the airway epithelium, their expression closely correlates with H_2_O_2_ release and key inflammatory cytokine production in CRSsNP and -wNP patients [15]. On the other hand, heme oxygenase (HO)-1, an enzyme proposed to be cytoprotective against oxidative stress, significantly increases in NP tissues compared to healthy controls [16]. More recently, it was demonstrated that the levels of p67^phox^ NADPH oxidase mRNA and protein, as well as 4-hydroxynonenal (4-HNE), are upregulated in NP tissue [17]. Lipopolysaccharide reduces cystic fibrosis transmembrane conductance regulator (CFTR)-mediated short-circuit current and increases cytoplasmic and mitochondrial reactive oxygen species, resulting in CFTR carbonylation in mammalian respiratory epithelial cells [18]. Interestingly, lectin-like, oxidized low-density lipoprotein (LDL) receptor-1, a major receptor for oxidized LDL produced by oxidative stress, is associated with disease severity in CRSwNP [19], and bactericidal antibiotics promote oxidative damage and programmed cell death in sinonasal epithelial cells [20]. While some special genes have been targeted in research on CRS, a systemic analysis of the transcriptional levels in oxidative-stress-related genes in nasal polyposis remains lacking.

In this study, we hypothesized that oxidative stress may be involved in the development and progression of CRSwNP. Therefore, the main goals of this study were to understand how nasal sinus mucosae and polyp tissues imbalance oxidant/antioxidant defense in CRSwNP development and severity by using a systemic transcriptomic analysis approach. Our results showed that an apparent increase in oxidative stress, including lipid peroxidation and protein nitrotyrosination, occurred in CRSwNP NP tissues, as compared to the controls. Of the 84 oxidative stress genes tested, 23 were differentially and significantly expressed, including 19 overexpressed and 4 underexpressed. The results were confirmed by a customized real-time PCR array and RT-PCR analysis as well as Western blotting. Further analysis suggested that LPO, MPO, SOD3, HO-1, and iNOS are possible endotype predictors for CRSwNP development.

## 2. Materials and Methods

### 2.1. Materials

The Abs raised against NOS2 (iNOS) was obtained from BD Biosciences (Becton Drive Franklin Lakes, NJ, MA, USA). The heme oxygenase-1 (HMOX1; HO-1) and superoxide dismutase 3 (SOD3) Ab were from Abcam (Cambridge, MA, USA). The Ab for GAPDH was purchased from GeneTex, Inc. (Hsinchu, Taiwan).

### 2.2. Patient Recruitment and Sample Collection

This study was approved by the Ethics Committee of the Shin Kong Wu Ho-Su Memorial Hospital, Taipei, Taiwan (Permission No: 20161210R) and conducted with the written informed consent of the patients. A total of 25 patients with CRSwNP and a control group of 25 patients with nasolacrimal duct obstructions were recruited. Briefly, CRSwNP was diagnosed based on criteria from EPOS 2020 [21]. The patients should have two symptoms, one of which should be nasal obstruction and/or discolored discharge ± facial pain/pressure ± reduction or loss of smell for more than 12 weeks. Furthermore, they should have either endoscopic signs of NPs, mucopurulent discharge primarily from the middle meatus, an edema/mucosal obstruction primarily in the middle meatus, and/or a CT scan showing mucosal changes within the ostiomeatal complex and/or sinuses. The staging of nasal polyposis was based on the Meltzer Clinical Scoring System, which is a 0–4 polyp grading system (0 = no polyps, 1 = polyps confined to the middle meatus, 2 = multiple polyps occupying the middle meatus, 3 = polyps extending beyond middle meatus, 4 = polyps completely obstructing the nasal cavity). None of the patients had been treated with oral or topical anti-allergic agents or steroids for at least 2 months. The patients’ characteristics, including ages, genders, and NP stage, are summarized in Supplementary Table S1. The 25 control patients comprised 17 males and 8 females; age: 38.8 ± 14.31 years old. The 25 patients with CRSwNP comprised 15 males and 10 females; age: 42.16 ± 17.49 years old. The NP score was 4.24 ± 1.665 (all of the above are presented with mean ± SD). The NP tissues were obtained through uncinectomy procedures and ethmoidectomy during functional endoscopic sinus surgery (FESS). In the control group, there were patients with blockages in their lacrimal drainage systems who were free of other nasal diseases. The agger nasi sinus cell mucosae were prepared during dacryocystorhinotomy procedures.

### 2.3. Real-Time PCR Microarrays

For balance in the sample availability, data acquisition, and statistical demand, seven randomly selected control and CRSwNP nasal tissue samples were used for real-time PCR microarray to catalog the full oxidative-stress-related gene expression. Total RNAs of human control nasal mucosae and NP tissues were isolated by using an RNeasy Mini kit (Qiagen, Valencia, CA, USA). The cDNA was transcribed by using an RT^2^ Reaction Ready First Strand Synthesis Kit (Qiagen) and was analyzed by using the human oxidative stress PCR array (Qiagen). The real-time PCR microarrays used an RT^2^ SYBR Green Fluor qPCR Mastermix (Qiagen) on a 7300 Real-Time PCR System (Applied Biosystems, Foster City, CA, USA). Data were normalized by using housekeeping genes (β-actin, glyceraldehyde-3-phosphate dehydrogenase (GAPDH), β2-microglobulin (B2M), ribosomal protein, large, p0 (RPLP0)) and analyzed by comparing the 2^−ΔΔCt^ of the normalized sample.

### 2.4. Confirmation and Validation of the Up- and Downregulated Genes by Customized Real-Time PCR Microarrays

To confirm and validate the differentially expressed genes observed in the microarrays, a customized human oxidative stress PCR array (Qiagen) was performed. Briefly, the randomly selected genes from the results of the first-round analysis, including unchanged GPX2, PRDX4, and DUSP1 genes and significantly regulated genes such as LPO, MPO, SOD3, NOS2, GCLM, HMOX1, and AKR1C2, were confirmed and verified. The specific primers for detecting the above gene expression and two housekeeping genes (β-actin and RPLP0) were custom-coated on the plate, and a similar procedure was performed as described in the real-time PCR microarray section. Data were normalized by using multiple housekeeping genes and analyzed by comparing the 2^−ΔΔCt^ of the normalized sample. In total, 18 additional human nasal tissue samples per group (control and CRSwNP group) were used for confirmation and validation.

### 2.5. Immunohistochemistry

The 4-hydroxynonenal (4-HNE) and 3-nitrotyrosine modifications and SOD1 expression in the control nasal mucosae and CRSwNP NPs were determined via immunohistochemistry (IHC), as previously described, with minor modifications [22]. Briefly, tissue sections were deparaffinized, and the slides were hydrated in graded ethanol before use. The sections were subsequently washed, immersed, and heated in a water bath for 20 min. The slides were incubated at 4 °C overnight with the primary specific Ab against 4-HNE, 3-nitrotyrosine, or superoxide dismutase 1 (SOD1) (Abcam) after being blocked with a buffer containing 10% FBS. The slides were then washed with TBS, incubated with Super Enhancer and Poly-HRP, and then developed with one-step 3-amino-9-ethylcarbazole (AEC) using the Super Sensitive Polymer-HRP IHC Detection System (Biogenex Laboratories, Inc., Fremont, CA, USA) for 5–30 min. Sections were counterstained in hematoxylin for 20–40 s, washed with tap water, and mounted with 100% glycerol. The quantitation of the staining results was performed using the Invitrogen Celleste 5.0 Image Analysis Software (Thermo Fisher Scientific, Waltham, MA, USA). The areas of positive staining for each patient’s 2 regions of interest (ROIs) in the captured images were identified by setting a cutoff value and computed according to the software instructions. The mean intensity of each ROI was calculated as the integrated optical density (OD) of all areas/total area sizes (pixel square) in an ROI. The mean intensity of positive staining was obtained by calculating the above intensity/total ROI number.

### 2.6. RT-PCR Analysis of mRNA Expression Level in Up- and Downregulated Genes

Changes in the mRNA expression levels in some of the significantly and differentially expressed genes observed in the microarrays were examined by using RT-PCR for individual transcripts. The tissues were processed as homogenates, and the primers used are listed in Table 1. The total RNA extraction, 1st strand cDNA synthesis, and PCR analysis were performed as previously described [23], except the annealing temperature for the PCR was set to 51–61 °C, depending on the sequences of the primers used.

### 2.7. Tissue Lysate Preparation and Western Blot Analysis

Nasal tissue lysates were prepared as previously described [24]. Total proteins were analyzed on SDS-polyacrylamide gels, electroblotted onto PVDF membranes, and then probed using a primary Ab. The immunoblots were developed using Immobilon Western Chemiluminescent HRP Substrate (EMD Millipore Corporation, Billerica, MA, USA). The membranes were stripped with a stripping buffer, washed, and reprobed with the Abs to examine the level of GAPDH and then developed.

### 2.8. Data Analysis

All data are expressed as the mean ± SEM unless otherwise indicated. Differences between groups were compared with an unpaired Student’s *t*-test. Differences were considered to be statistically significant at *p* < 0.05. Logistic regression analysis and receiver operating characteristic (ROC) curves were performed to determine the ability of oxidative gene expression to distinguish the development of CRSwNP. The cutoff value was obtained based on the ROC curve analysis. The statistical analyses were performed using SPSS statistics software version 19.0 (IBM, Chicago, IL, USA).

## 3. Results

### 3.1. The Oxidative Damage Is Increased in the NPs of CRSwNP

**Figure 1:**
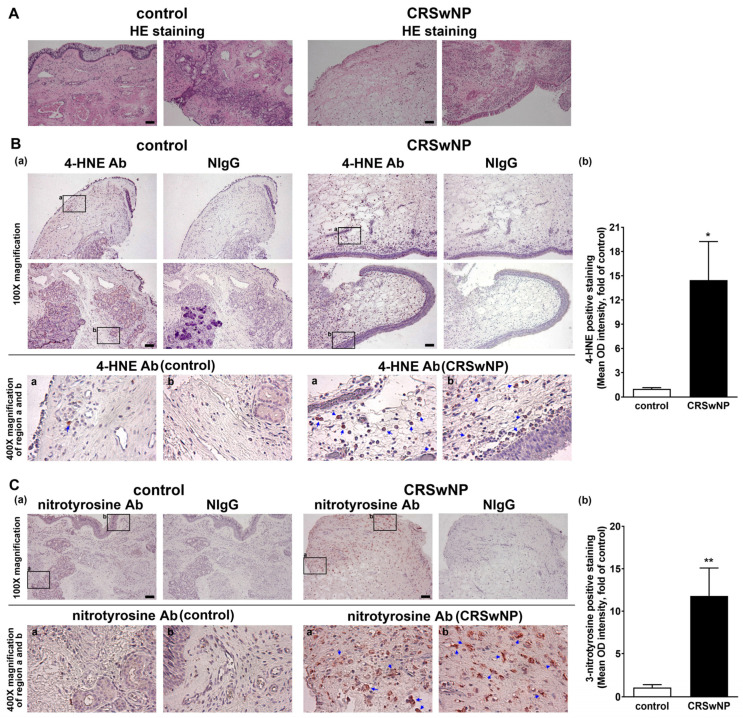
IHC analysis of 4-HNE and 3-nitrotyrosine expression levels in nasal tissues. The control nasal mucosae and NPs were stained by (**A**) hematoxylin and eosin (HE) staining and (**B**) anti-4-HNE Ab or (**C**) 3-nitrotyrosine Ab and its corresponding nonimmune IgG (NIgG). The selected regions (black rectangle frames (**a**,**b**) were magnified and images were captured under a microscope. The images shown below indicate positive staining, in which the deep red color spots/areas indicate the presence of 4-HNE or 3-nitrotyrosination (blue arrows) (panel (**a**)). A quantitative analysis of similar data for 4-HNE (*n* = 6) and 3-nitrotyrosine (*n* = 3) staining is also shown (panel (**b**)). Scale bar = 100 μm. * *p* < 0.05 and ** *p* < 0.01 versus control.

Polyunsaturated fatty acids are susceptible to peroxidation, and they yield various degradation products, including main α, β-unsaturated hydroxyalkenal, and 4-hydroxy-2,3-trans-nonenal (4-HNE), in oxidative stress [25]. In addition, 3-nitrotyrosine is a promising biomarker of oxidative stress formed due to the nitration of protein-bound and free tyrosine residues by reactive peroxynitrite molecules [26]. To investigate the role of oxidative stress in the NPs of CRSwNP, we assessed the lipid and protein markers of oxidative stress, including both 4-HNE and 3-nitrotyrosine modifications, using immunohistochemistry (IHC). In Figure 1A, hematoxylin and eosin staining (HE staining) was performed to indicate the location of the nucleus and cytoplasm/matrix proteins in the nasal mucosae and NP tissues. Next, in Figure 1B, the presence of any lipid peroxidation product, namely, 4-HNE, was evaluated. The two adjacent tissue slides were stained by the specific Ab for 4-HNE and its corresponding nonimmune IgG (NIgG). Little 4-HNE was found in the control nasal mucosae, but a lot of positive staining for 4-HNE (deep red color) was detected in the NP tissues of CRSwNP (blue arrows in the enlarged regions). The positive staining appeared to be located in (subepithelial) stroma cells and infiltrated leukocytes underneath the epithelium, as judged by comparing them to their corresponding cell’s morphology/shape in NIgG images. Meanwhile, protein markers of oxidative stress, 3-nitrotyrosine modifications, were also assayed using IHC. The positive staining, representing protein tyrosine nitrosylation, was more apparent and diffused in the NP tissues, as compared to the controls. The positive staining was located in the infiltrated leukocytes but was expressed in a more diffused fashion, which was not confined to the subepithelium region (Figure 1C). Overall, the quantitative analysis revealed a significant and marked increase in the positive staining of 4-HNE and 3-nitrotyrosine in the NPs of CRSwNP (Figure 1B,C, right panels), indicating a substantial increase in oxidative stress in CRSwNP NP tissues.

### 3.2. A Transcriptional Profile of Genes Involved in Oxidative Stress in CRSwNP NP Tissues

To investigate the impact of the changes observed in the NPs of patients with CRSwNP on the transcriptional profile of genes involved in oxidative stress, we applied quantitative real-time PCR microarrays to catalog gene-expression levels in a total of 84 genes in the control nasal mucosae and the NPs of patients with CRSwNP. Genes were considered to be expressed differentially and significantly in the NPs of patients with CRSwNP if the expressions were greater than a two-fold change, with a *p*-value of >0.05. As detailed in Table 2, of the 84 genes tested, 23 were differentially and significantly expressed (19 were overexpressed (blue color text/number) and 4 were underexpressed (red color text/number)). Although the changes in some genes in the NP group, such as PRDX4, DUOX1/DUOX2, and DUSP1, were more than two-fold, they did not reach statistical significance.

**Figure 2:**
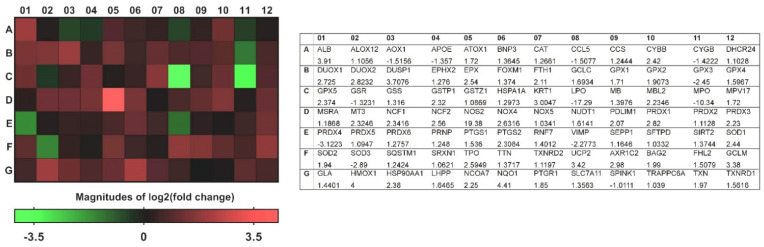
Visualization of fold changes in oxidative stress gene expression between the control and CRSwNP groups for each gene in the array in the context of an array layout. The heat map and table provide fold regulation data used for the map as well as abbreviations for the genes associated with each datum.

**Figure 3:**
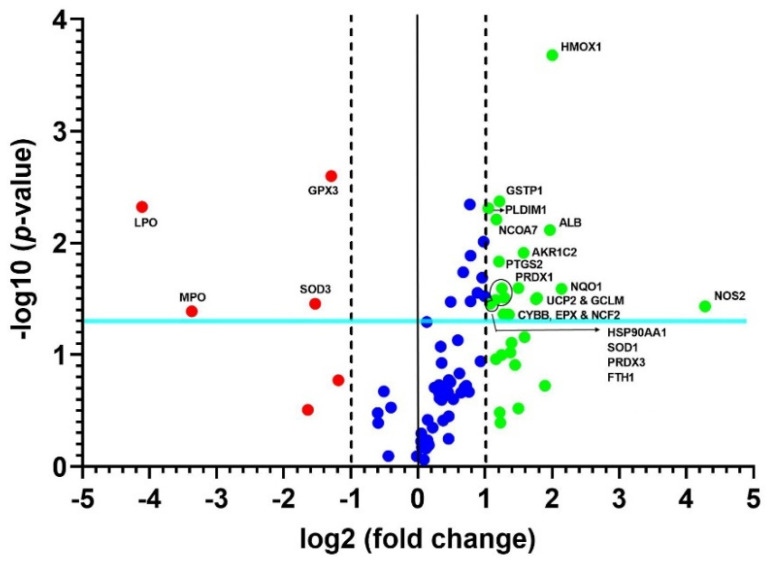
Volcano plot of changes in significant oxidative stress gene expressions. The 84 genes in the human nasal mucosae and NPs associated with oxidative stress were analyzed. The volcano plot displays statistical significance versus fold changes on the *y*- and *x*-axes, respectively. The *x*-axis represents fold changes from the control, whereas the *y*-axis represents the *p*-values. The vertical solid line in the middle and the other two vertical dashed lines indicate a one-fold change and a threshold of two-fold changes in gene expression, respectively. Note that the light blue line indicates a *p*-value of 0.05. The significantly changed genes are located outside the two vertical dashed lines and above the blue line, and they are labeled with their respective gene names.

A heat map of the 84 genes provided an overall visualization of the fold changes in their expressions between the control and CRSwNP groups for each gene in the array in the context of an array layout (Figure 2). It was found that the two pronouncedly upregulated genes were the inducible nitric oxide synthase (iNOS; NOS2) and heme oxygenase-1 (HMOX1; HO-1), whereas the two more downregulated genes were LPO and MPO. The changes were more than three-fold, comprising seven upregulated genes (ALB, NOS2, UCP2, GCLM, HMOX1, NQO1, and AKP1C2) and two downregulated genes (LPO and MPO). The significantly regulated genes in CRSwNP that had a greater than two-fold change with a *p*-value > 0.05 could be easily observed in a volcano plot (Figure 3).

### 3.3. Confirmation and Validation of the Significantly Changed Genes

**Figure 4:**
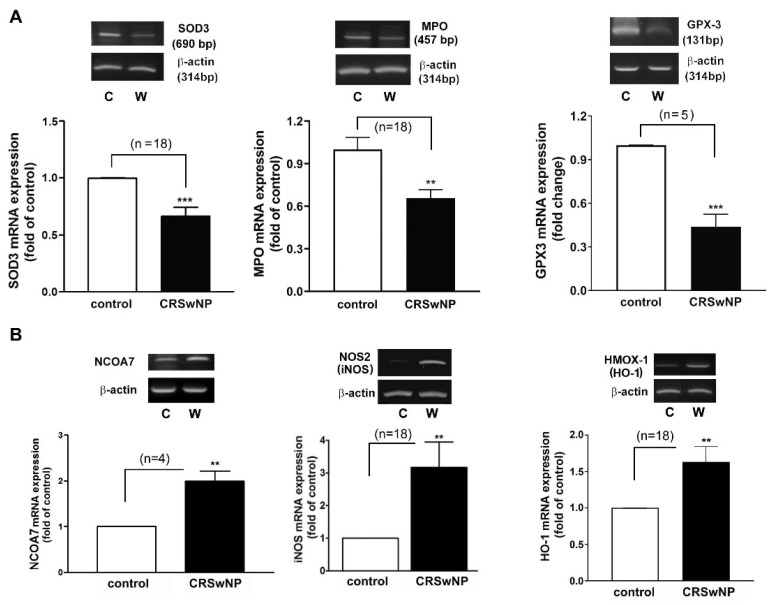
Verifying the expression level of the representative changed genes using RT-PCR analysis. The total RNA was extracted from human control nasal mucosae and CRSwNP NP tissues. (**A**) The representative downregulated GPX3, SOD3, and MPO and (**B**) the upregulated NCOA7, NOS2 (iNOS), and HMOX-1 (HO-1) mRNA expression levels were determined using RT-PCR to verify their changes in a real-time PCR array assay. Data are mean ± SEM. C: control; W: CRSwNP. ** *p* < 0.01 and *** *p* < 0.001 versus control.

Next, an RT-PCR analysis of the selected individual transcripts of the genes was performed. The data confirmed the significant regulation of mRNA expression in iNOS (3.1-fold increase), NCOA7 (2-fold increase), HMOX1 (also called HO-1) (1.633-fold increase), GPX3 (2.1-fold decrease), SOD3 (1.5-fold decrease), and MPO (1.5-fold decrease) in the CRSwNP NPs (Figure 4).

The significantly and differentially changed genes observed in the microarrays were further confirmed and validated using a customized real-time PCR array analysis on the 18 additional patients’ nasal tissue samples using probes targeting significantly and non-significantly changed genes. Three non-significantly changed genes, including GPX2, PRDX4, and DUSP1, were selected, whereas seven significantly changed genes, including LPO, MPO, SOD3, NOS2, GCLM, HMOX1, and AKR1C2, were chosen from Table 2. In Table 3, the customized real-time PCR array analysis revealed that the three non-significantly changed genes remained non-significantly unchanged. Consistently, the significantly changed LPO, MPO, SOD3, NOS2 (iNOS), GCLM, HMOX1 (HO-1), and AKR1C2 gene expressions in the 18 additional CRSwNP NP testing samples were confirmed and kept at significantly changed levels. The *p*-values for six of the seven validated genes were smaller than 0.001. Based on the results shown in Table 3, the changing levels of LPO, MPO, SOD3, and NOS2 (iNOS) were more than three-fold compared to the control.

**Figure 5:**
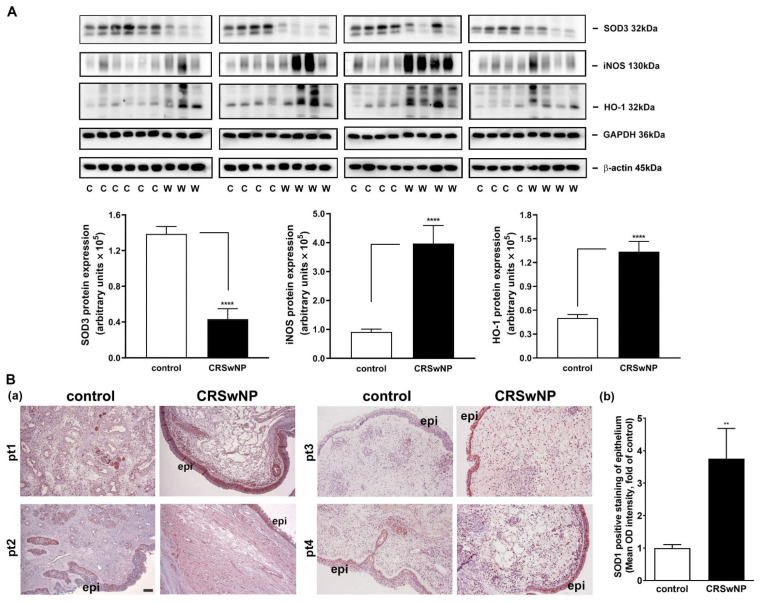
Western blot and IHC analysis of protein expression levels in the representative altered genes in the control nasal mucosae and CRSwNP NPs. (**A**) The protein levels of the representative altered genes in the control nasal mucosae and CRSwNP NPs, including SOD3, iNOS (NOS2), and HO-1, were analyzed in 18 human nasal control mucosae and 15 CRSwNP NP tissues with Western blotting. The iNOS was analyzed with WB in a nonreduced form. A quantitative analysis of the results was performed using densitometry. Data are mean ± SEM. **** *p* < 0.0001 versus control. (**B**) (**a**) An IHC analysis of SOD1 expression in four representative control nasal mucosae and NP tissues from the control and CRSwNP patients, respectively. Note that the positive staining was mainly found in the epithelium, which is quantified and shown in panel (**b**) (*n* = 6). pt: patient; epi: epithelium. Scale bar = 100 μm. ** *p* < 0.01 versus control.

We confirmed the mRNA levels in selected changed genes in the CRSwNP NPs, including iNOS, NCOA7, HMOX1, GPX3, SOD3, and MPO (Figure 4). To further investigate whether the up- and downregulated gene products, i.e., proteins, are correspondingly increased in the NPs of CRSwNP, a Western blot analysis was performed. The representative gene-encoded proteins in the 18 nasal mucosae of the controls and the 15 NPs of the CRSwNP patients (due to running out of the 3 NP samples) were analyzed with Western blotting. In Figure 5A, a markedly reduced expression of the SOD3 protein was noted in the CRSwNP NPs, and an approximately 2.5-fold reduction was found. On the other hand, robust, increased levels of both NOS2 (iNOS) and HMOX-1 (HO-1) in the NPs of CRSwNP were observed, as compared to the control: about 4.4- and 2.66-fold increases, respectively. Moreover, the enhanced positive staining of SOD1 was observed mainly in the epithelium and, partly, in the subepithelial regions via an IHC assay (Figure 5B(a)). A quantitative analysis of the epithelium regions revealed a significantly increased positive staining intensity in the NP tissues (panel b). These results demonstrated that the proteins of the changed genes were also simultaneously altered in the NPs.

### 3.4. The Oxidative Gene Expression Level and Its Association with the Development of CRSwNP

**Figure 6:**
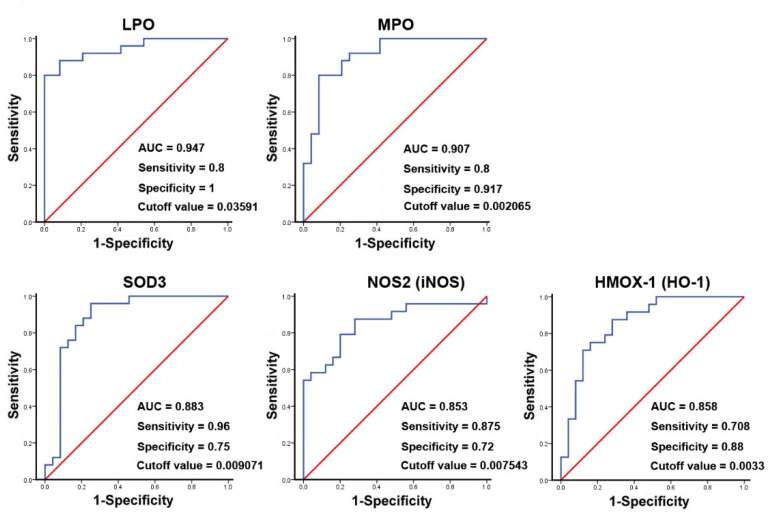
ROC curve analysis of potential oxidative stress biomarkers used to predict the development of CRSwNP. The markedly changed genes, including LPO, MPO, SOD3, NOS2 (iNOS), and HMOX-1 (HO-1), in the NPs of 25 patients with CRSwNP were analyzed using an ROC curve analysis. The *p*-values for the genes were 8.32 × 10^−8^, 1.06 × 10^−6^, 4.22 × 10^−6^, 2.24 × 10^−5^, and 1.71 × 10^−5^, respectively.

The binary logistic regression analysis for the significant genes shown in the results of Table 3 demonstrated that the changed genes were associated with CRSwNP endotypes (data not shown). Therefore, a ROC curve analysis was then performed. The result suggested that the area the under the curve (AUC) values for LPO, MPO, and SOD3 were 0.947, 0.907, and 0.883, whereas, for NOS2 (iNOS) and HMOX-1 (HO-1), they were 0.853 and 0.858, respectively. The specificity and sensitivity for LPO were 1 and 0.8 and for MPO were 0.917 and 0.8, respectively. In contrast, the specificity and sensitivity for SOD3 were 0.75 and 0.96, and for iNOS, they were 0.72 and 0.875, respectively. These indicated that LPO, MPO, and HO-1 have a stronger ability to distinguish true negative rates, whereas SOD3 and iNOS have a relatively good ability to find the true positive rates for CRSwNP at a certain optimized cutoff value (Figure 6).

## 4. Discussion

As oxygen (O_2_) is mandatory for energy production in human cells, it may also contribute to the production of oxidizing free radicals [27]. Low levels of ROS production are required to maintain physiological functions such as signal transduction and host defense, but a higher level of ROS due to an imbalance between oxidant and antioxidant defense systems can produce oxidative stress, damaging cellular macromolecules [28,29]. To date, there has been no in vivo systemic analysis to determine the pathways that cause damage due to oxidative stress in CRSwNP. In this study, we demonstrated that the distribution and expression of 4-hydroxynonenal (4-HNE) and 3-nitrotyrosine as markers of oxidative stress—which is represented by lipid peroxidation and the protein nitration of tyrosine residues in CRSwNP NPs—were more severe than those found in control nasal mucosae. More importantly, of the 84 genes examined using quantitative real-time PCR microarrays, we found that 19 genes were significantly upregulated and 4 genes were downregulated in CRSwNP NPs as compared to the control (Table 2). Confirmation and validation processes using the customized PCR array (Table 3), RT-PCR (Figure 4), and Western blotting (Figure 5) further confirmed and demonstrated the specificity and sensitivity of the results of the oxidative stress PCR array. The overall reaction from the up- and downregulation of oxidative-related genes leads to a substantial increase in oxidative stress (Figure 1).

Nitric oxide (NO) synthase 2, also known as inducible NO synthase (iNOS), was the most significantly upregulated gene. NOS2 is well known for catalyzing L-arginine to produce nitric oxide (NO), which is a free radical that acts as a cellular signaling molecule in many different biochemical processes [30]. In the respiratory tract, NO is involved in both type I and type II immune responses, in which type I inflammation and type II immune cell proliferation are activated/associated with low levels of NO and IgE production/higher NO concentrations, respectively [31,32]. Markedly increased iNOS gene expression and protein expression appear to be associated with type II inflammation in CRSwNP nasal polyposis. A previous study showed that the expression of iNOS in both epithelial and stromal layers was consistently greater in NP than in middle turbinate tissues. Moreover, the allergic NP group showed more iNOS expression than the non-allergic NP group [33].

Members of the oxidative stress-responsive gene family, including eosinophil peroxidase (EPX/EPO), glutamate–cysteine ligase, modifier subunit (GCLM), ferritin, heavy polypeptide 1 (FTH1), heme oxygenase (decycling) 1 (HMOX1), PDZ and LIM domain 1 (PDLIM1), superoxide dismutase 1, soluble (SOD1), and NAD(P)H dehydrogenase (NQO1) were all upregulated in the real-time PCR array assay. NQO1, also known as NAD(P)H quinone oxidoreductase, was the most significantly upregulated gene identified by the microarray analysis. It is an antioxidant flavoprotein that reduces quinones to hydroquinones and indirectly prevents the one-electron reduction of quinone to the semiquinone free radical [34,35]. The enzyme is mainly regulated by the ARE (antioxidant response element) under normal and oxidative stress conditions [36]. In a glioblastoma study, the knockdown of NQO1 augmented ROS and diminished cell proliferation, whereas the overexpression of NQO1 attenuated ROS and increased cell proliferation in glioblastoma cells [37]. Therefore, it is suspected that NQO1 upregulation possibly reflects polyp tissue proliferation during nasal polyposis. On the other hand, HMOX1/HO-1, an enzyme proposed to be cytoprotective against oxidative stress, is also significantly increased in NP tissues compared with healthy controls, as observed by others [16]. There was a study showing that DUOX1 and -2 mRNA are significantly upregulated in CRSsNP and -wNP [15]. As to this observation, they were increased in our study but did not reach significance. Regarding the SODs, it is known that each SOD has a distinct subcellular localization but catalyzes the same reaction. The distinct location of these SOD isoforms is particularly important for compartmentalized redox signaling, mainly including the dismutation of superoxide to generate H_2_O_2_ and, subsequently, H_2_O via catalase [38]. Interestingly, SOD1 (CuZnSOD), a soluble and intracellular form of SOD, increased in NPs, whereas the mitochondrial SOD2 (MnSOD) was without change, and SOD3 (ecSOD), an extracellular (secreted) form of SOD, decreased in NPs (Table 2 and Table 3 and Figure 4 and Figure 5). Since SOD3 is mainly located within the extracellular matrix (ECM) and on the cell surface and binds ECMs such as proteoglycan and collagen, it is suspected that a decrease in extracellular SOD3 may lead to an increase in O_2_^•-^ in extracellular space. However, an increase in cellular SOD1 may have an inverse effect on the dismutation of superoxide in intracellular cytoplasm. In this regard, it has been reported that SOD-1 and -2 decrease and that SOD-3 is unchanged in (non)eosinophilic CRSwNP. Moreover, SOD activity decreases [39]. Since the reduction in SOD-3 expressed in NPs in our study was evaluated using a PCR array, RT-PCR, and Western blotting, we can confirm here that this finding is very convincing. The discrepancies are unknown and remain to be investigated. It was recently reported that thioredoxin (TRX)-interacting protein (TXNIP) may have a negative association with TRX, and the decreased SOD activities and increased malondialdehyde levels resulting in the upregulation of ROS and oxidative stress in nasal epithelial cells may play a pivotal role in the pathogenesis of CRSwNP [40]. Therefore, although the expression in SOD isoforms could vary in our and other studies, it is suspected that overall SOD activity decreases in CRSwNP.

In this study, one of the striking findings was the identification of a significant and marked reduction in LPO and MPO expression in CRSwNP. LPO, MPO, and EPX/EPO all belong to heme peroxidases, which contribute to immune protection on epithelial surfaces and in secretions [41]. LPO is known to be a major antimicrobial protein found in milk, saliva, tears, and airway secretions that works with H_2_O_2_ from dual oxidases (DUOX) to produce antimicrobial substances. LPO uses H_2_O_2_ and thiocyanate (SCN^−^) substrate to generate antimicrobial hypothiocyanite (OSCN^−^) [41,42]. Therefore, the downregulation of LPO subsequently compromising innate immune defense may explain, at least in part, why many microbiomes are involved in the pathogenesis of CRSwNP. Consistently, MPO in neutrophils and, to a lesser extent, in monocytes [43] also derives reactive oxidant hypochlorous acid (HOCl), playing an important role in neutrophil antimicrobial activity and human defense [43,44]. It was reported that the dense inflammatory infiltrate of the typical histological features of NPs is composed of several immune cell types, such as T and B lymphocytes, group 2 innate lymphoid cells, eosinophils and neutrophils, mast cells, and macrophages [45]. Thus, it was supposed that MPO should be upregulated in CRSwNP NPs. However, the results were the opposite. CRSwNP has long been considered an eosinophil-dominant Th2 inflammatory disease, but in Eastern Asian populations, >50% of CRSwNP cases are non-eosinophil-dominant and, in some samples, neutrophils are the dominant cell type, characterized by mixed Th1 or Th17 type inflammation [46,47]. Thus, a markedly reduced MPO expression in NPs may reflect a higher possibility of eosinophilic CRSwNP in Taiwanese people. This could be supported by our observation that the EPX/EPO expression mainly expressed in eosinophils was elevated in NPs (Table 2). There was also a study showing that MPO promotes the development of lung neutrophilia and indirectly influences subsequent chemokine and cytokine production by other cell types in the lung, so the attenuation of MPO expression is notedly decreased in lung injuries [48]. Moreover, MPO may form part of a negative feedback loop that downregulates inflammation, limits tissue damage, and facilitates the switch from innate to adaptive immunity in the physiological setting of acute, neutrophil-mediated inflammation [49]. Taken together, the decrease in LPO and MPO may reduce the production of HOXs such as HOCl, HOSCN, HOBr, and HOI and, thus, compromise the antimicrobial activity of the nasal epithelium.

Regarding the functional consequence of changed genes in CRSwNP, this results in the enhancement of oxidative stress, leading to the formation and production of protein tyrosine nitrosylation and 4-HNE in NPs (Figure 1), which could contribute to nasal polyposis. A decrease in SOD3 may cause an increase in O_2_^•−^ accumulation and a decrease in H_2_O_2_ in extracellular space. Moreover, an apparent decrease in LPO and MPO and a lack of H_2_O_2_ sources may lead to a reduction in OSCN^−^ production, which may compromise the microbicidal ability of the nasal epithelium. Meanwhile, the reaction between O_2_^•−^ and nitric oxide (NO^•^) may produce ONOO^−^, whose decomposition, in turn, gives rise to some highly oxidizing intermediates, including NO_2_^•^, OH^•^, and CO_3_^•−^, as well as, ultimately, stable NO_3_^−^ [38,50]. The nitric oxide and peroxynitrite molecules contribute to protein tyrosine nitrosylation [26], which was observed in our study (Figure 1). On the other hand, numerous initiators of lipid peroxidation, such as 4-HNE in biological systems, are often hydroxyl radical (OH^•^), ozone (O_3_), nitrogen oxide (NO) and dioxide (NO_2_), and sulfur dioxide (SO_2_) [51]. This may result from the downregulation of LPO, MPO, and SOD3. In addition, the upregulation of PRDX1 and -3, CYBB, PTGS2 (COX-2), NCF2, and NOS2 (iNOS) may contribute to O_2_^•−^ and NO production. We also found that the increase in free radicals was accompanied by an increase in antioxidant systems such as NOQ1, preventing the one-electron reduction of quinone to the semiquinone free radical GCLM (known as gamma-glutamylcysteine synthetase) from participating in the first-rate limiting step of glutathione synthesis, as well as preventing GSTP1 from detoxifying reduced glutathione.

Previous studies generally focused on the relationship between control and CRS nasal tissues with respect to selected oxidative-stress-related genes, such as iNOS, HO-1, SOD, PRDX, and others [14,15,39,52]. In this study, we systemically analyzed NP tissues using 84 preset oxidative-stress-related genes, targeting and providing a whole analytic picture of up- and downregulated genes that might contribute to the generation of oxidative stress, including lipid peroxidation and protein nitrotyrosination during nasal polyposis. Nevertheless, there were some limitations in this study. Due to limited sample size, expenses, and sample manipulations, the sample number in the first-round screening and statistical analysis of the 84 genes was confined to seven, and only some of the genes could be verified in the following customized microarray, RT-PCR, Western blot, and IHC analyses. Moreover, because of the limited sample size and time, the study did not divide the CRSwNP patients into primary and recurrent subgroups, and, therefore, we could not find differences in the expression of oxidative stress genes between them. Since recurrent CRSwNP is also an important issue in this field [53], in the future, further analysis of these data and an increase in the sample size in both subgroups could identify a potential predictive marker for recurrent CRSwNP.

## 5. Conclusions

Some researchers have suggested that antioxidants, such as glutathione, N-acetylcysteine, polyphenols, vitamin C, etc., could be used to treat upper airway inflammation [54,55]. The gene changes in this study show the imbalance between oxidative stress and antioxidant defense in CRSwNP progression. Several oxidative stress genes, such as NOS2 and HMOX1, were upregulated, which indicates the presence of oxidative stress. Meanwhile, some antioxidant genes, such as LPO, MPO, GPX3, and SOD3, were downregulated, leading to the impairment of antioxidant defense. The imbalance substantially increased oxidative stress and the formation of 4-HNE and 3-nitrotyrosine products in CRSwNP NPs. The summary of our findings is illustrated as a schematic diagram (Supplementary Figure S1). Currently, there are only a few endotype classifications for CRS. While acknowledging the limited gene profiling in CRSwNP patients, our comprehensive and systemic analysis of oxidative-stress-related gene expression reveals, for the first time, novel evidence of the potential involvement of specific genes in redox regulation regarding the development and progression of CRSwNP. Further research is required to explore the mechanisms and pathophysiological significance of our findings in CRSwNP.

## Figures and Tables

**Table 1:** Primers for reverse transcription polymerase chain reaction.

Gene	Forward Primer (5′→3′)	Reverse Primer (5′→3′)	Product Size
*SOD3*	ATGCTGGCGCTACTGTGTT	GCTTCTTGCGCTCTGAGT	690 bp
*GPX3*	GCCGGGGACAAGAGAAGT	GAGGACGTATTTGCCAGCAT	131 bp
*MPO*	CGCCAACGTCTTCACCAATG	GATGTCGATGTTGTTGGGCG	457 bp
*NCOA7*	GCCACACTTCTCACTGCTCA	TAGGACAGGCAGCACCTCTT	181 bp
*iNOS*	GCAGAATGTGACCATCATGG	ACAACCTTGGGGTTGAAGGC	426 bp
*HMOX1*	GAGACGGCTTCAAGCTGGTGATG	GTTGAGCAGGAACGCAGTCTTGG	500 bp
*β-ACTIN*	ATCATGTTTGAGACCTTCAA	CATCTCTTGCTCGAAGTCCA	314 bp

**Table 2:** Eighty-four oxidative-stress-related gene expressions in the NPs of patients with CRSwNP (the up- and downregulated genes with a *p*-value < 0.05 are highlighted in blue and red, respectively).

Gene Ref No.	Gene Name	Fold Regulation	*p*-Value
NM_000581	Glutathione peroxidase 1 (GPX1)	1.71	0.00453
NM_002083	Glutathione peroxidase 2 (gastrointestinal) (GPX2)	1.9073	0.11468
NM_002084	Glutathione peroxidase 3 (plasma) (GPX3)	−2.45	0.00253
NM_002085	Glutathione peroxidase 4 (phospholipid hydroperoxidase) (GPX4)	1.5967	0.01834
NM_001509	Glutathione peroxidase 5 (epididymal androgen-related protein) (GPX5)	2.374	0.10027
NM_000637	Glutathione reductase (GSR)	−1.3231	0.29685
NM_000178	Glutathione synthetase (GSS)	1.316	0.18993
NM_000852	Glutathione S-transferase pi 1 (GSTP1)	2.32	0.00425
NM_001513	Glutathione transferase zeta 1 (GSTZ1)	1.0869	0.68955
NM_001979	Epoxide hydrolase 2, cytoplasmic (EPHX2)	1.276	0.25403
NM_002574	Peroxiredoxin 1 (PRDX1)	2.82	0.02537
NM_005809	Peroxiredoxin 2 (PRDX2)	1.1128	0.62476
NM_006793	Peroxiredoxin 3 (PRDX3)	2.23	0.0324
NM_006406	Peroxiredoxin 4 (PRDX4)	−3.1223	0.31044
NM_181652	Peroxiredoxin 5 (PRDX5)	1.0947	0.05096
NM_004905	Peroxiredoxin 6 (PRDX6)	1.2757	0.11847
NM_001752	Catalase (CAT)	1.2661	0.08469
NM_000397	Cytochrome b-245, beta polypeptide (CYBB)	2.42	0.04344
NM_134268	Cytoglobin (CYGB)	−1.4222	0.21308
NM_175940	Dual oxidase 1 (DUOX1)	2.725	0.1233
NM_014080	Dual oxidase 2 (DUOX2)	2.8232	0.30205
NM_004417	Dual specificity phosphatase 1 (DUSP1)	3.7076	0.1896
NM_000502	Eosinophil peroxidase (EPX)	2.54	0.04358
NM_006151	Lactoperoxidase (LPO)	−17.29	0.00476
NM_000250	Myeloperoxidase (MPO)	−10.34	0.04083
NM_000962	Prostaglandin-endoperoxide synthase 1 (prostaglandin G/H synthase and cyclooxygenase) (PTGS1)	1.536	0.14707
NM_000963	Prostaglandin–endoperoxide synthase 2 (prostaglandin G/H synthase and cyclooxygenase) (PTGS2)	2.3084	0.01467
NM_000547	Thyroid peroxidase (TPO)	2.5949	0.09569
NM_003319	Titin (TTN)	1.3717	0.5637
NM_000477	Albumin (ALB)	3.91	0.0077
NM_000041	Apolipoprotein E (APOE)	−1.357	0.80674
NM_005954	Metallothionein 3 (MT3)	2.3246	0.32864
NM_080725	Sulfiredoxin 1 (SRXN1)	1.0621	0.86638
NM_003330	Thioredoxin reductase 1 (TXNRD1)	1.5616	0.21785
NM_006440	Thioredoxin reductase 2 (TXNRD2)	1.1197	0.64345
NM_005410	Selenoprotein P, plasma, 1 (SEPP1)	1.1646	0.4487
NM_000454	Superoxide dismutase 1, soluble (SOD1)	2.44	0.03099
NM_000636	Superoxide dismutase 2, mitochondrial (SOD2)	1.94	0.02045
NM_003102	Superoxide dismutase 3, extracellular (SOD3)	−2.89	0.03504
NM_000697	Arachidonate 12-lipoxygenase (ALOX12)	1.1056	0.38209
NM_005125	Copper chaperone for superoxide dismutase (CCS)	1.2444	0.21894
NM_000265	Neutrophil cytosolic factor 1 (NCF1)	2.3416	0.40425
NM_000433	Neutrophil cytosolic factor 2 (NCF2)	2.56	0.04391
NM_000625	Nitric oxide synthase 2, inducible (NOS2)	19.38	0.037
NM_016931	NADPH oxidase 4 (NOX4)	2.6316	0.07836
NM_024505	NADPH oxidase, EF-hand calcium-binding domain 5 (NOX5)	1.0341	0.50355
NM_003355	Uncoupling protein 2 (mitochondrial, proton carrier) (UCP2)	3.42	0.03109
NM_001159	Aldehyde oxidase 1 (AOX1)	−1.5156	0.33213
NM_004052	BCL2/adenovirus E1B 19 kDa-interacting protein 3 (BNIP3)	1.3645	0.21708
NM_002437	MpV17 mitochondrial inner membrane protein (MPV17)	1.72	0.01303
NM_003019	Surfactant protein D (SFTPD)	1.0332	0.59598
NM_004045	ATX1 antioxidant protein 1 homolog (yeast) (ATOX1)	1.72	0.03331
NM_002985	Chemokine (C-C motif) ligand 5 (CCL5)	−1.5077	0.4059
NM_014762	24-dehydrocholesterol reductase (DHCR24)	1.1028	0.58043
NM_021953	Forkhead box M1 (FOXM1)	1.374	0.35409
NM_002032	Ferritin, heavy polypeptide 1 (FTH1)	2.11	0.03497
NM_001498	Glutamate–cysteine ligase, catalytic subunit (GCLC)	1.6934	0.21463
NM_002061	Glutamate–cysteine ligase, modifier subunit (GCLM)	3.38	0.03198
NM_002133	Heme oxygenase (decycling) 1 (HMOX1)	4	0.00021
NM_005345	Heat shock 70 kDa protein 1A (HSPA1A)	1.2973	0.38672
NM_006121	Keratin 1 (KRT1)	3.0047	0.06958
NM_000242	Mannose-binding lectin (protein C) 2, soluble (MBL2)	2.2346	0.10945
NM_012331	Methionine sulfoxide reductase A (MSRA)	1.1868	0.19668
NM_000903	NAD(P)H dehydrogenase, quinone 1 (NQO1)	4.41	0.02578
NM_002452	Nudix (nucleoside diphosphate linked moiety X)-type motif 1 (NUDT1)	1.6141	0.19677
NM_020992	PDZ and LIM domain 1 (PDLIM1)	2.07	0.0049
NM_183079	Prion protein (PRNP)	1.248	0.24491
NM_014245	Ring finger protein 7 (RNF7)	1.4012	0.03359
NM_203472	Selenoprotein S (VIMP)	−2.2773	0.16892
NM_012237	Sirtuin 2 (SIRT2)	1.3744	0.16827
NM_003900	Sequestosome 1 (SQSTM1)	1.2424	0.18601
NM_003329	Thioredoxin (TXN)	1.97	0.00976
NM_005368	Myoglobin (MB)	1.3976	0.17586
NM_001354	Aldo-keto reductase family 1, member C2 (dihydrodiol dehydrogenase 2; bile acid-binding protein; 3-alpha hydroxysteroid dehydrogenase, type III) (AKR1C2)	2.98	0.0123
NM_004282	BCL2-associated athanogene 2 (BAG2)	1.99	0.03
NM_001450	Four and a half LIM domains 2 (FHL2)	1.5079	0.07401
NM_000169	Galactosidase, alpha (GLA)	1.4401	0.24986
NM_001017963	Heat shock protein 90 kDa alpha (cytosolic), class A member 1 (HSP90AA1)	2.38	0.0255
NM_022126	Phospholysine phosphohistidine inorganic pyrophosphate phosphatase (LHPP)	1.6465	0.18936
NM_181782	Nuclear receptor coactivator 7 (NCOA7)	2.25	0.00618
NM_012212	Prostaglandin reductase 1 (PTGR1)	1.85	0.02804
NM_014331	Solute carrier family 7 (anionic amino acid transporter light chain, xc- system), member 11 (SLC7A11)	1.3563	0.22685
NM_003122	Serine peptidase inhibitor, Kazal type 1 (SPINK1)	−1.0111	0.8077
NM_024108	Trafficking protein particle complex 6A (TRAPPC6A)	1.039	0.67335

**Table 3:** Confirmation of the selected gene expressions in Table 2 via a customized microarray analysis of 18 additional NPs from the CRSwNP patients; up- and downregulated genes with *p*-values < 0.05 are highlighted in blue and red, respectively.

No.	Gene Name	Fold Regulation	*p*-Value
1	Glutathione peroxidase 2 (gastrointestinal) (GPX2)	1.2269	0.53943
2	Peroxiredoxin 4 (PRDX4)	−1.6239	0.00016
3	Dual specificity phosphatase 1 (DUSP1)	−1.1024	0.8687
4	Lactoperoxidase (LPO)	−16.662	<1 × 10^−6^
5	Myeloperoxidase (MPO)	−3.5766	0.00017
6	Superoxide dismutase 3, extracellular (SOD3)	−3.2295	0.000095
7	Nitric oxide synthase 2, inducible (NOS2)	5.6641	0.024785
8	Glutamate–cysteine ligase, modifier subunit (GCLM)	2.0229	0.000028
9	Heme oxygenase (decycling) 1 (HMOX1)	2.3368	0.000017
10	Aldo-keto reductase family 1, member C2 (dihydrodiol dehydrogenase 2; bile acid-binding protein; 3-alpha hydroxysteroid dehydrogenase, type III) (AKR1C2)	2.3061	0.000751

## Data Availability

The data are contained within the article and supplementary materials.

## Supplementary Materials

The following supporting information can be downloaded at: https://www.mdpi.com/article/10.3390/antiox11101899/s1, Table S1: Patients’ characteristics. Figure S1: The proposed schematic diagram of changed genes involved in the generation of oxidative stress in CRSwNP.
