# Supplementary material for: Transcriptomic Analysis of Genes Associated with Oxidative Stress in Chronic Rhinosinusitis Patients with Nasal Polyps: Identifying Novel Genes Involved in Nasal Polyposis
Source: Antioxidants (Basel). 2022 Sep 25;11(10):1899. doi: 10.3390/antiox11101899 (PMC9598890; doi:10.3390/antiox11101899)
Supplement: Supplementary file 1 [file antioxidants-11-01899-s001.zip › antioxidants-1888442-supplementary.pdf]

Supplementary Materials

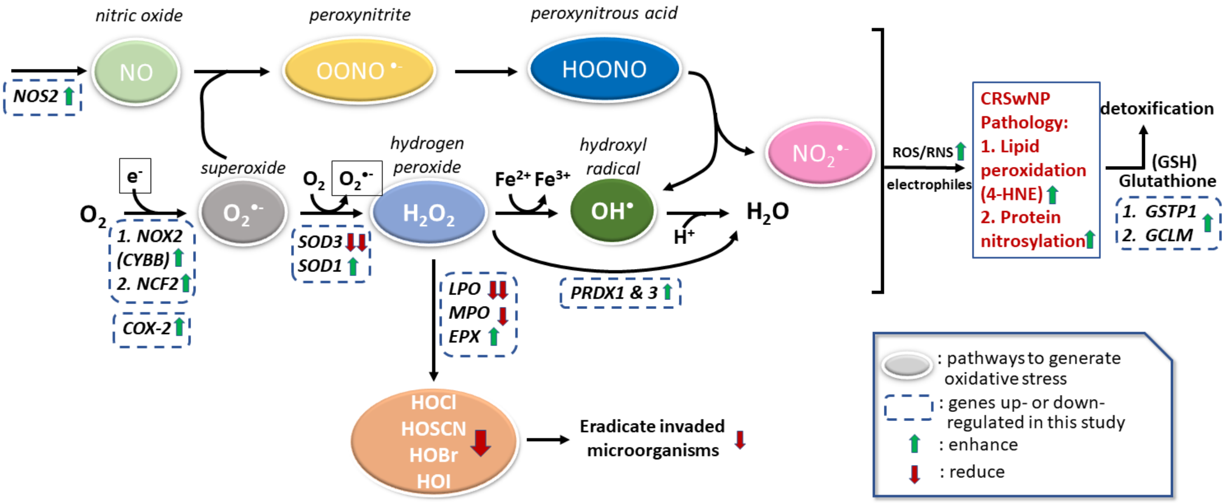

Figure S1. The proposed schematic diagram of some changed genes involved in generation of oxidative stress in nasal polyposis of CRSwNP. The reactive oxygen/nitrogen species (ROS/RNS) and electrophiles generated through oxidative stress and other xenobiotic pathways lead to lipid peroxidation and protein nitrosylation of nasal tissues. Moreover, the marked decrease in LPO and MPO may decrease the production of the corresponding HOXs (hypochlorous acid (HOCl), hypothiocyanous acid (HOSCN), hypobromous acid (HOBr) and hypoiodous acid (HOI)) and result in an increase of invaded microorganisms. The identified gene products in the dash-line box may participate in this process, where the accompanied up and down arrows indicate that gene is up- or down-regulated in nasal polyps (NPs). These final oxidative products may orchestrate to cause lipidation and protein tyrosine nitrosylation in nasal tissues during nasal polyposis of CRSwNP pathology.

Table S1. Patients' characteristics.

	**Control (n = 25)**	**CRSwNP (n = 25)**
**Age (years, min-max)**	38.8 ± 14.31 (17–74)	42.16 ± 17.49 (18–76)
**Sex (m/f)**	17/8	15/10
**Nasal polyp score (sum of left and right nostril scores, min-max)**	-	4.24 ± 1.665 (2–6)
